# An evaluation of the benefits and harms of antenatal corticosteroid treatment for women at risk of imminent preterm birth or prior to elective Caesarean-section: Study protocol for an individual participant data meta-analysis

**DOI:** 10.12688/wellcomeopenres.15661.1

**Published:** 2020-02-25

**Authors:** Elizabeth Wastnedge, Joshua Vogel, Jasper V. Been, Cynthia Bannerman-Gyamfi, Ewoud Schuit, Devender Roberts, Rebecca M. Reynolds, Sarah Stock, Co_Opt collaboration

**Affiliations:** 1 Centre for Reproductive Health, University of Edinburgh, Edinburgh, UK; 2 Maternal and Child Health Program, Burnet Institute, Melbourne, Australia; 3 Division of Neonatology, Department of Paediatrics, Division of Obstetrics and Gynaecology and Department of Publisc Health, Erasmus MC, Rotterdam, The Netherlands; 4 Department of Obstetrics and Gynaecology, Columbia University, New York, USA; 5 Julian Center for Health Sciences and Primary Care, University Medical Center Utrecht, Utrecht, The Netherlands; 6 Liverpool Women's Hospital NHS Foundation Trust, Liverpool, UK; 7 Centre for Cardiovascular Science, University of Edinburgh, Edinburgh, UK

**Keywords:** Preterm birth, Antenatal corticosteroids, Respiratory distress syndrome.

## Abstract

**Background: **Antenatal corticosteroid treatment (ACT) has been widely accepted as a safe, beneficial treatment which improves outcomes following preterm birth. It has been shown to reduce respiratory distress syndrome and neonatal mortality and is commonly used in threatened or planned preterm delivery, as well as prior to elective Caesarean-section at term. There are some concerns however, that in some cases, ACT is used in patients where clinical benefit has not been established, or may potentially increase harm. Many women who receive ACT do not deliver preterm and the long-term consequences of ACT treatment are unclear. This study aims to evaluate the benefits and harms of ACT using latest trial evidence to allow refinement of current practice.

**Methods: **This study will compare ACT with placebo or non-treatment. Inclusion criteria are: Randomised Controlled Trials (RCT) comparing ACT vs. no ACT (with or without placebo) in all settings. Exclusion criteria are: non-randomised or quasi-randomised studies and studies comparing single vs. multiple courses of ACT. Main outcomes are to evaluate, for women at risk of preterm birth or undergoing planned Caesarean- section, the benefits and harms of ACT, on maternal, fetal, newborn, and long-term offspring health outcomes.

The individual participant data (IPD) of identified RCTs will be collected and consecutively synthesised using meta-analysis with both a one-stage model where all IPD is analysed together and a two-stage model where treatment effect estimates are calculated for each trial individually first and thereafter pooled in a meta-analysis. Sub-group analysis will be performed to identify heterogeneous effects of ACT across predefined risk groups.

**Discussion: **Co-opt is the Consortium for the Study of Pregnancy Treatments and aims to complete a robust evaluation of the benefits and harms of ACT. This IPD meta-analysis will contribute to this by allowing detailed interrogation of existing trial datasets.

**PROSPERO registration:** CRD42020167312 (03/02/2020)

## List of abbreviations

ACT - Antenatal corticosteroid treatment

ALPS – Antenatal Late Preterm Steroids trial

BMI – Body Mass Index

CS – Caesarean section

CPAP – Continuous Positive Airways Pressure

ECMO – Extra-corporeal Membrane Oxygenation

GA - Gestational age

HPA axis – Hypothalamic-Pituitary-Adrenal axis

IPD - Individual participant data

IVH - Intraventricular Haemorrhage

LMIC - Low- and middle-income countries

NEC - Necrotising enterocolitis

NICU – Neonatal Intensive Care Unit

PIH – Pregnancy Induced Hypertension

PPROM – Preterm prelabour rupture of membranes

PRISMA-IPD - Preferred Reporting Items for Systematic review and Meta-analysis using Individual Patient Data

PRISMA-P - Preferred Reporting Items for Systematic review and Meta-analysis Protocols

PVL – Periventricular leukomalacia

RCT - Randomised controlled trial

RDS – Respiratory Distress Syndrome

UN - United Nations

WHO - World Health Organisation

## Plain language summary

When babies are born prematurely, respiratory problems are the most common cause of illness and death and the most common reason for admission to the special care baby unit or neonatal intensive care. Babies born by planned Caesarean-section are also at greater risk of respiratory problems than those born by vaginal delivery.

Antenatal corticosteroid treatment (ACT) has long been used to help reduce respiratory problems and neonatal death and is a cornerstone of treatment when preterm birth (defined as birth at <37 weeks gestation ^1^) is planned or expected, or when Caesarean-section at any gestation, is planned. Corticosteroids are produced naturally by the mother when she goes into labour. Giving corticosteroids to the mother if she is at risk of giving birth prematurely, aims to augment this natural response. Giving ACT to the mother helps the lungs of the fetus to mature more quickly, to enable them to function better after birth.

Although ACT has been used widely for a number of years, the evidence of benefit from randomised controlled trials is based mainly on those who receive ACT at 26-34 weeks of pregnancy. While there are observational data showing benefit at other gestational ages, there is some concern that the benefit may not be as great as previously estimated, particularly in later gestation, and there may even be risk of harm. The evidence suggests greatest benefit from ACT is derived if they are given between 24 hours and 7 days before delivery, however timing of preterm birth is very difficult to predict. This means many women are being given ACT but then do not give birth within 7 days. In these women it is uncertain whether potential benefit outweighs the potential harms of ACT.

The risk profile of ACT is not fully understood. It is known that the short term risks of ACT include increased rates of neonatal hypoglycaemia (low blood sugar), which if not recognised and treated appropriately is an independent risk factor for developmental delay ^2– 5^. There is a lack of evidence assessing the long-term risks. However there is concern that being exposed to ACT as a fetus could increase risk of conditions such as cardiovascular disease and diabetes mellitus later in life ^2^. With these risks in mind, it is important to fully establish the risk benefit profile for all babies, and their mothers, receiving ACT.

This study will collate data from previous trials with the aim of gaining greater understanding of the potential benefits and harms of ACT in mothers and in babies born at all gestations and by all modes of delivery, and also in those who receive ACT but do not deliver within 7 days. The results will be used to inform clinical guidelines on ACT use and allow clinicians to follow best evidence-based practice.

## Introduction

### Description of the condition

Preterm birth is the leading cause of death in newborns, and is responsible for 35% of neonatal mortality ^6^. Respiratory distress syndrome (RDS) is one of the main causes of early neonatal mortality and morbidity in premature neonates. It affects one third of babies born before 32 weeks and is caused primarily by a combination of immature lung development, surfactant deficiency and immaturity in other organ systems ^7^.

### Description of the intervention

ACT mimics the natural surge of endogenous corticosteroids which occurs around term in women who labour, and works by thinning alveolar walls to increasing lung surface area for gas exchange ^3^. It also causes increased transcription of surfactant by type II pneumocytes which increases tissue compliance and lowers surface tension ^3, 8^. It was first evaluated in humans in Liggins’ seminal randomised-controlled trial (RCT) in 1972 ^9^, and this evidence was consolidated by a systematic review in 1990 ^10^. Since then the usage of ACT has increased worldwide. In the USA, between 1991-1999, ACT use increased from 24%–72% in preterm births, and in California a cross-sectional analysis of 33,610 low birth weight babies between 2005–2011, usage was as high as 92.9% ^11^

ACT has been shown to confer significant morbidity and mortality benefit for both preterm neonates, and neonates born by elective Caesarean section who do not receive the same physiological endogenous surge ^3^. It reduces RDS rates overall, as well as reducing moderate and severe RDS and need for respiratory support for all neonates ^3^. For preterm neonates it also has a vasoconstrictive effect on the cerebral blood flow, thereby reducing rates of intraventricular haemorrhage ^3, 7^. ACT also reduces necrotising enterocolitis (NEC) and rates of retinopathy of prematurity, as well as early systemic infection and requirement for neonatal intensive care unit (NICU) admission ^7, 12^.

ACT is widely used and widely accepted as a safe and beneficial treatment for preterm labour, planned preterm birth and elective Caesarean-section. It is given when preterm labour is predicted or a preterm Caesarean-section is planned, to improve fetal lung maturation, and confer other morbidity and mortality benefits ^2, 7, 13^. ACT (specifically dexamethasone phosphate) has been listed on the WHO Model List of Essential Medicines ^14^ as well as being identified as one of the UN 13 life-saving commodities for mother and child ^15, 16^.

There is however, a lack of evidence of the long-term consequences of ACT. There is concern that excess exposure to ACT as a fetus may alter Hypothalamic-Pituitary-Adrenal (HPA) axis programming and predispose to increased risk of metabolic disease with dyslipidaemia, impaired glucose metabolism and hypertension, in addition to behavioural changes such as attention deficit, increased aggression or elevated stress response ^2, 17^.

Trial data on long-term consequences is limited, although one 30 year follow up of trial data found no significant difference in body size, blood lipids, blood pressure, plasma cortisol or cardiovascular disease between the group who had received ACT and the control group, although they did find increased fasting insulin which is an early marker of insulin resistance ^18^. From observational studies, data collected on body size, blood pressure and behavioural assessment from children who had received ACT at two year follow up found no significant differences in anthropometric or neurocognitive measures ^19, 20^. There is however clear evidence from animal data that exposure to glucocorticoid excess during pregnancy predisposes to adverse metabolic outcomes in adult life ^17, 21^.

### Current evidence and knowledge

There have been three recent Cochrane reviews evaluating the use of ACT vs. placebo for preterm birth in different circumstances ^7, 22, 23^. The largest: “Antenatal corticosteroids for accelerating fetal lung maturation for women at risk of preterm birth”, updated by Roberts *et al.* in 2017, comprises 30 RCTs including 7774 women and 8158 infants assessing the use of ACT in acceleration of fetal lung maturation among women with imminent preterm delivery ^7^. The review authors concluded ACT is associated with a reduction in a number of adverse outcomes related to prematurity including perinatal death, neonatal death and moderate/severe RDS ^7^. A separate review in 2018 evaluated ACT use compared to placebo in Caesarean-section at term: “Corticosteroids for preventing neonatal respiratory morbidity after elective Caesarean-section at term”. This review included 4 trials with a total of 3956 women and 3893 live infants and again found a reduction in all RDS (Risk ratio (RR) 0.48 95% Confidence Interval (CI) 0.27-0.84) ^22^. A 2015 Cochrane review “Repeat doses of prenatal corticosteroids for women at risk of preterm birth” assessed the risks and benefits of repeat courses of ACT if birth does not occur within 7 days of first dose ^23^. This included 10 trials with 4733 women and 5700 infants who had received one course of ACT, and compared those who had further courses, with those who had no further treatment. Short term benefits were seen with reduction in RDS (RR 0.83, 95% CI 0.75-0.91) in women who had multiple courses, and there was no increase in adverse outcomes seen with repeat courses ^23^. Subsequent to this, an IPD meta-analysis has been completed evaluating repeat courses and identified 11 trials ^24^. They too found reduction in need for respiratory support (RR0.91, 95% CI 0.85-0.97) and no significant difference in serious outcome (RR 0.92, 95% CI 0.82-1.04) ^24^. A systematic literature evaluating six clinical trials which included 5698 women receiving steroids at greater than 34 weeks gestation, found that in this group RDS was significantly reduced (RR 0.74; 95% CI 0.61-0.91). Neonatal care requirements were also reduced with less mechanical ventilation, surfactant administration and NICU admission ^25^.

Recently, the Antenatal Corticosteroids Trial triggered significant concerns into current ACT usage. It was a large, multi-centre, cluster-randomised trial published in 2015 by Althabe *et al*., designed as a implementation trial evaluating a package of interventions to increase the use of ACT in 6 LMICs for women at risk of PTB. The trial included data from 51 intervention clusters including 48219 women and 48698 births and 50 control clusters including 51523 women and 52007 births. Although the study was successful in increasing ACT uptake, there was minimal evidence of benefit in small infants (<5 ^th^ birthweight percentile), evidence of harm in larger infants (>25 ^th^ birthweight centile), and increase in maternal infection rates ^26, 27^. These findings were surprising and led to further evaluation of the existing evidence

### ACT use in specific patient groups

***ACT given >34 weeks gestation.*** In the Roberts *et al*. review ^7^ there was a post-hoc sub-group analysis of ACT use among women greater than 34 weeks gestation. In this subgroup they found ACT did reduce RDS (RR 0.65; 95% CI 0.58-0.73) but there was no significant reduction in perinatal mortality (RR 1.03; 95% CI 0.29-3.67) ^7^. The Antenatal Late Preterm Steroids (ALPS) trial contributed much of these data, and did also find that ACT use was associated with increased rates of neonatal hypoglycaemia in the treatment group (RR 1.60; 95% CI, 1.37 to 1.87) ^5^.

***ACT given prior to planned Caesarean-section.*** Neonates delivered by Caesarean section are known to have higher rates of respiratory morbidity than those born vaginally, and these rates are higher still in planned Caesarean-section before the onset of labour ^21, 22, 28^. Although some of this increase is due to the indication for Caesarean-section, the RDS in these neonates may have a different pathophysiology to that in prematurity and the lack of the physiological corticosteroid surge which occurs during labour is a contributing factor ^22^. As discussed above, the Cochrane review found a 52% reduction in RDS following planned Caesarean-section when ACT was given 48 hours before ^22^. However, there was considerable risk of bias in the evidence as three of the four studies included were unblinded. In addition, there was no significant change in mortality, (RR 0.67, 95% CI 0.11-4.10). Given that in high income settings there are now facilities to manage neonatal RDS, if ACT has long-term health implications risks may outweigh the benefits in this sub-group ^21, 29^.

***Time from ACT to delivery >7 days.*** The cellular changes caused by ACT are acute and transitory, and there is some evidence that ACT is beneficial when given 24 hours to 7 days before delivery, but that after 7 days there is no benefit ^3, 30^. However, even when threatened preterm labour is established, it is not possible to accurately estimate exactly when delivery will occur. It has been reported that up to a quarter of women who were given ACT did not deliver within 7 days ^3, 31^. There is limited data on ACT use in this sub-group and we are unsure of the benefits and possible harms for these women.

If after 7 days of ACT administration, birth has not occurred (but is still considered imminent) current guidelines recommend repeating ACT ^32^. The Cochrane review evaluating repeat doses of ACT found a reduction in RDS (RR 0.83, 95% CI 0.75-0.91) and serious infant outcome (RR 0.84, 95% CI 0.75-0.94) following a repeat course of ACT ^23^. There was no evidence at childhood follow-up (24 months) of any statistically significant differences in rates of neurodisability (including cerebral palsy and cognitive impairment) (RR 1.03, 95% CI 0.71-1.50). Further long-term follow up however is required to evaluate the long-term benefits and risks for both women and babies ^24^.

***ACT given to those who deliver at term.*** It is difficult to accurately predict when birth will occur after threatened preterm labour ^33^. The ORACLE collaborative group conducted a trial of treatment of 4826 women with Preterm Prelabour Rupture of Membranes (PPROM) and found 80% of women “diagnosed” with preterm labour actually delivered after 37 weeks ^34^. While there is evidence that ACT given to women at greater than 37 weeks’ gestation in the context of elective Caesarean-section reduces incidence of RDS and thereby reduces neonatal care requirements ^25^, this short term benefit may not be worthwhile in the context of possible long-term risk.

### How the intervention might work

In late gestation and during labour, there is a surge of endogenous corticosteroids ^3^, and ACT administration aims to imitate this. This surge is important in the development of multiple organ systems and supports the transition from the intra- to extra-uterine environment. In the lungs, corticosteroids stimulate the transcription of surface proteins involved in surfactant production, and also play a key part in alveolar fluid clearance post-delivery ^3, 35^. In addition, corticosteroids act systemically in both the mother and the fetus, affecting the brain, heart, kidneys, hypothalamus and circulation ^3^.

### Why it is important to do this review

With the clear evidence of short-term benefit of ACT, particularly in preterm babies, few may question the rationale for continued usage. However the lack of evidence on long term effects of ACT, particularly among those receive ACT but are not born preterm, mean there are further questions to be answered. By performing an IPD analysis we will be able to look at all published trial data on ACT and perform more detailed sub-group analysis with the aim of answering questions on those who receive ACT but do not deliver within 7 days, those who receive ACT but deliver at term, and also the differential benefits of ACT at different gestational ages. This study will be complemented by an IPD analysis using observational data, evaluating the same outcomes, as well as giving the opportunity to evaluate long-term outcomes from cohort data (PROSPERO CRD42019137260).

Out of the 30 studies included in the Roberts *et al*. review, 14 were published prior to 1990 ^7^. Neonatal care has seen significant advances during that time- notably the use of continuous positive airways pressure (CPAP), and the availability of surfactant therapy. It may be that when compared with current standard neonatal care within a high income setting, ACT no longer produces such significant reductions in mortality and morbidity ^2, 21^. In the USA there were significant reductions in infant mortality from RDS prior to the widespread introduction of ACT ^4^. With this in mind, it is possible that current ACT practice may be conferring insufficient benefit to the neonate to justify the exposure to unquantified long-term risks of metabolic disease and neurocognitive alteration ^2, 17, 21^ By performing subgroup analysis of year of birth on IPD, we will be able to glean greater understanding about the absolute impact of ACT in the context of current neonatal care. In addition, from our initial scoping searched, since the Roberts et al. Cochrane review, there have been four new ACT RCTs published ^36– 39^, as well as an additional four studies currently in progress. This means that in addition to the detailed sub-group analysis we will be able to perform with the IPD approach, we will also have a body of new data to add to the picture.

In addition, there was considerable variability in the inclusion of women with high-risk conditions such as preterm prelabour rupture of membranes (PPROM). Using aggregate data it is hard to evaluate the differential effects on these women whereas IPD analysis will allow a more nuanced approach to identifying individual women with specific high-risk conditions ^40^.

This study aims to aims to answer some of the outstanding questions around the risks and benefits of ACT use, by using an IPD approach. It has a number of benefits such as the ability to quality assess data in greater detail and to standardise outcomes and statistical methods across studies ^40^. It will also allow more detailed sub-group analysis to enable exploration of the effects of ACT among specific patient groups, such as in multiple pregnancies, or in maternal infection. This is difficult to do with aggregate data and we will be able to provide a more nuanced analysis. We are also able to account for the impact of other interventions, such as tocolysis ^41, 42^. IPD is also able to produce more clinically relevant results than aggregate data analysis alone, as it enables more powerful assessment of treatment effects ^40^. The current ACT usage is a “one-size fits all” approach and the ambition of the IPD meta-analysis is to breakdown the appropriateness of this with greater granularity than an aggregate data approach allows.

### Aims

The aims of this study are to undertake a robust evaluation of the benefits and harms of ACT given to women at risk of preterm birth. We aim to ascertain, with greater specificity than previous meta-analyses, the impact of ACT use at different gestational ages. We also aim to evaluate with greater specificity what the effective dose is, and the effective time window between administration and subsequent delivery. This evaluation will help refine the criteria for ACT administration to maximise benefits and avoid potentially harmful side effects.

### Objectives

***Primary objective.*** To evaluate, for women at risk of preterm birth or undergoing planned Caesarean-section, the benefits and harms of ACT compared to placebo or no treatment, on maternal, fetal, newborn, and long-term offspring health outcomes.

## Methods and design

### Protocol development and registration

This protocol has been registered with PROSPERO ( CRD42020167312) on 3 February 2020, and has been developed in accordance with the Preferred Reporting Items for Systematic review and Meta-analysis Protocols (PRISMA-P) checklist (please see reporting guidelines) ^43^.

### Inclusion and exclusion criteria

Inclusion criteria: RCTs comparing ACT vs. no ACT (with or without placebo) in all settings.

Exclusion criteria: non-randomised or quasi randomised studies. Women and infants participating in studies looking at single vs multiple courses of ACT.

### Population

Women with singleton or multiple pregnancy at any gestation, who receive ACT as part of a RCT trialling ACT for preterm labour or elective Caesarean-section at any gestation.

### Intervention

Antenatal administration of any exogenous corticosteroid commonly used for fetal lung maturation given by IM injection (betamethasone or dexamethasone). Any dosing regimen will be accepted.

### Comparators

The comparator trial arm- women at risk of preterm birth or prior to elective Caesarean-section at any gestation who receive either placebo, or no treatment.

### Study design

RCTs will be included. Non-randomised or quasi-randomised trials will be excluded to minimise bias. Where IPD cannot be obtained, aggregate data from the study will be included. Sensitivity analysis will be performed to account any impact of this.

### Trial identification

Initial literature searches and screening have been carried out as part of the development of this protocol. Roberts 2017 Cochrane review of all RCTs evaluating ACT use ^7^ was used as a baseline and the Cochrane Pregnancy and Childbirth search strategy was used as the basis of our strategy and re-run to include papers published since their search completed in June 2017. Databases searched (as per the Cochrane strategy) were MEDLINE, Embase, CINAHL and Cochrane. Search strategies are provided as extended data ^44^. In addition to this, the trial registers ( ClinicalTrials.gov, ISCTRN and the WHO ICTRP portal) were also searched in order to identify any relevant ongoing trials, and any unpublished trials where data collection had been completed were eligible for inclusion. Reference lists of review papers and other relevant studies were also screened for relevant papers.

All titles and abstracts identified by the search will be screened independently by two reviewers to identify full papers. If no full paper is available, authors will be contacted. Any discrepancies in screening will be resolved by discussion with a senior group member.

Details of the screening including reasons for inclusion and exclusion will provided as a PRISMA diagram.

### Data provision and coding

Trial investigators will be asked to submit data in a standardised, anonymised format using standardised coding developed for this project. If this is not possible, anonymised data will be accepted in any reasonable format and re-coded by the research team.

Data will be requested for all women randomised for the trial, including any who were excluded from the trial analysis.

All patient identifiable information including identifying numbers will be removed and replaced by a sequential numbering system which will be kept securely by the research group.

A list of data items to be requested can be found in the extended data ^44^.

### Data storage and confidentiality

A data management plan will be developed prior to receipt of the data. All IPD will be received via secure online transfer or encrypted email. It will be stored securely on the University of Edinburgh server. Data will be accessible only to those working directly on the project. No data will be copied to personal devices such as memory sticks or laptops.

### Critical appraisal, data checking and quality assurance

The data will be critically appraised based on the trial protocols, the publications and on checking of the IPD. Risk of bias will be assessed using the Cochrane Revised risk-of-bias tool (ROB2) ^45^. At least two researchers will undertake quality assessment of data with any discrepancies to be resolved by a senior member of the group.

All IPD data will be checked on receipt for consistency and integrity of randomisation. Data will be compared with the trial publication for any inconsistencies. Again this will be done by two independent researchers. In case of inconsistencies, the principal investigator of the trial will be contacted.

If any datasets are deemed to be insufficient quality (based on a “High” ROB2 score), they will be excluded from the analysis. This will be done either for the dataset as a whole, or from particular analyses, depending on the data problem.

### Data description

A descriptive table along with a narrative summary will be produced outlining the key design features and demographic characteristics of each dataset included. Excluded datasets will be listed along with reasons for exclusion.

### Main outcomes

***Primary fetal/neonatal outcomes.*** Extended perinatal mortality (defined as stillbirth or death within first 28 days of life ^46^)

***Primary maternal outcome.*** Maternal infection up to 6 weeks after trial entry (chorioamnionitis, pyrexia requiring antibiotics, puerperal sepsis, intrapartum fever requiring antibiotics or postnatal pyrexia requiring antibiotics)

***Primary long-term outcomes for offspring.*** Neurodevelopmental disability at follow-up (blindness, deafness, moderate/severe cerebral palsy (however defined by study authors), or development delay/intellectual impairment (defined as developmental quotient or intelligence quotient less than -2 standard deviation below population mean), or cerebral palsy (abnormal tone with motor dysfunction)).

Adverse cardiometabolic outcomes at follow up (lipid dysregulation, high blood pressure (as defined by NICE CKS) ^47^, impaired glucose tolerance (based on oral glucose tolerance test), presence of type 2 diabetes (based on fasting glucose or glucose tolerance test).

### Secondary outcomes

The following additional outcomes will be explored where data allows. Where possible we have aligned other outcomes to those of both the Roberts *et al.* Cochrane review ^7^, and also the Crown group core outcomes set for interventions to prevent preterm birth ^48^.

***Secondary fetal/neonatal outcomes.***

1. Stillbirth
2. Respiratory Distress Syndrome
3. Moderate/severe respiratory distress syndrome
4. Birthweight (g)
5. Hypoglycaemia (blood glucose <2.5mmol/l)
6. Neonatal Unit Admission (level and duration)
7. Neonatal infection confirmed by positive culture (early (within first 72 hours) and late)
8. Chronic lung disease (need for continuous supplemental oxygen at 28 days postnatal age or 36 weeks’ postmenstrual age, whichever was later)
9. Apgar score less than seven at five minutes
10. Intraventricular haemorrhage (IVH) grade 3 or 4
11. Periventricular leukomalacia (PVL)
12. Retinopathy of prematurity grade 3 or 4
13. Interval between trial entry and birth
14. Length at birth (height)
15. Head circumference at birth
16. Small-for-gestational age (<3rd centile for gestational age by WHO 1990 birthweight standards)
17. Necrotising enterocolitis (Stage 2 or 3)

***Secondary maternal outcomes.***

1. Mortality
2. Admission to intensive care unit
3. Side effects of therapy (gastrointestinal upset, glucose intolerance or pain/infection at injection site)
4. Postnatal depression

### Sensitivity and supplementary analysis

Sensitivity and subgroup analyses will be used to explore differences in characteristics, datasets and alternative approaches to data synthesis. We will analyse potential effect modifiers to identify any particular patient groups who derive greater benefit or harm from ACS administration. We will also investigate whether particular therapeutic regimens are more effective than others, if data are available. All analyses will be described according to whether they were principle, subgroup or sensitivity analyses and whether they were pre-planned or post hoc.

If sufficient data exists, we will perform subgroup analyses on the following groups for the primary outcomes:

1. Number of fetuses in utero (singleton or multiple birth)
2. Indication for administration of ACT
3. Hypertensive disorders of pregnancy (pre-eclampsia, gestational, chronic) vs no hypertensive disorders of pregnancy
4. PPROM vs no PPROM
5. Suspected fetal growth restriction (FGR) vs no FGR
6. Type of glucocorticoid (betamethasone or dexamethasone)
7. GA when first course of ACT given (<23 weeks, 23–28 weeks, 28–32 weeks, 32–34 weeks, 34–36 weeks, >36 weeks)
8. Length of time from administration of first dose of ACT until birth (<48 hours, 48 hours-7 days, 7–14 days, >14 days.)
9. Diabetes mellitus (including gestational and pre-gestational diabetes) vs no diabetes mellitus
10. Concurrent tocolytic use vs no tocolytic use
11. Country income level (High, middle or low as defined by The World Bank ^49^)
12. Ethnicity (white vs African/Caribbean vs Asian vs mixed vs other)
13. Sex of baby

We will perform the following sensitivity analyses on the primary outcomes to evaluate the impact of trial design on the results:

1. High rate of loss to follow up
2. Year of birth (pre- and post- year 2000 and the era of modern neonatal care)
3. Trails with a high risk of bias (as measured using the RoB II tool ^45^)
4. Inclusion of aggregate data where IPD not available

## Statistical methods

### Planned analysis

A detailed statistical analysis plan will be developed in discussion with a statistician when the extent of available data is known, before starting the analysis. The analysis will be performed on an intention to treat basis.

### Outcome measures

For dichotomous outcomes, a log-binomial regression model will be performed to calculated risk ratios comparing ACT use with placebo/no treatment. For continuous outcomes, linear regression will be performed to calculate mean differences between treatment arms. For time-to-event outcomes, cox regression will be performed to calculate hazard ratios.

### Unit of analysis

Pregnancy will be used as the unit of analysis for maternal and birth outcomes and liveborn child will be used for infant and childhood outcomes. Analyses will be adjusted for clustering (for example with Generalised Estimating Equations) in multiple pregnancies where possible.

### One- and two-stage models

We will use one-stage models- where all IPD from all trials is analysed together accounting for clustering within trials. We will also use two-stage models where effect estimates are calculated for each trial individually then pooled in a meta-analysis.

### Subgroup analysis

Subgroup analysis in one-stage meta-analysis will be performed by adding an interaction term between the subgrouping variable and treatment allocation to the regression model. If there is a sign of interaction (p _interaction_ < 0.1), one-stage meta-analysis will be stratified by the subgrouping variable.

In two-stage meta-analysis, stratified analysis by the subgrouping variable will be performed on the trial level and results will be pooled in a meta-analysis. Heterogeneity of the stratified treatment effects will be assessed to determine whether any effect modification is present.

### Relative and absolute differences

Absolute differences and number needed to treat will be calculated by applying risk ratios to baseline incidences.

### Unavailable trials and missing data

If we are unable to obtain IPD from a given study, aggregate data from the publication will be used where possible and be incorporated via two-stage meta-analysis.

Datasets in which any particular outcome or variable is not recorded will not contribute to related analyses. Where data are missing for some participants, a complete case analysis excluding these patients will be performed initially. If there are more substantial missing data (>10% for any covariate), multiple imputation will be used to impute missing variables within each dataset. Sensitivity analyses will be used to assess the impact of missing outcome data.

### Software

Analysis will be performed using the R software package.

### Reporting

Results will be presented and discussed with the Co-opt group, with whom the interpretation of results and final report will be confirmed. Results will be reported in concordance with the PRISMA-IPD ^50^. Plain language summaries of findings will be produced.

### Data repository

This analysis will result in the creation of a new database of IPD stored securely and anonymously. This data will only be shared with the explicit approval of data controllers. Without this approval, data will be securely destroyed at the end of the project within the secure network. If data sharing is permitted we will create and anonymised data sharing repository.

### Dissemination of information

We intend to publish our findings in a peer-reviewed scientific journal. We also intend to present any findings at relevant scientific conferences. All aggregate data will be collated and shared upon publication and where permissions are given we will share all IPD.

### Study status

A systematic literature search has been completed by two independent reviewers and we are currently in the process of contacting authors of all identified studies to invite them to collaborate.

## Discussion

This IPD meta-analysis is part of a wider project by the Co-opt working group evaluating the use and effects of medications during pregnancy. All investigators of this analysis are part of the Co-opt group and all results will be discussed by the group. Results will be reported in accordance with the PRISMA-IPD guidelines and will be used to inform evidence-based clinical practice.

## Data availability

### Underlying data

No data are associated with this article.

### Extended data

Open Science Framework: An evaluation of the benefits and harms of antenatal corticosteroid treatment for women at risk of imminent preterm birth or prior to elective Caesarean-section: an individual participant data meta-analysis. https://doi.org/10.17605/OSF.IO/2TQNK ^44^

This project contains the following extended data:

- Data outcomes.docx (List of data items to be collected)
- Search strategies.docx (Study search strategies)

### Reporting guidelines

Open Science Framework: PRISMA-P checklist for ‘An evaluation of the benefits and harms of antenatal corticosteroid treatment for women at risk of imminent preterm birth or prior to elective Caesarean-section: Study protocol for an individual participant data meta-analysis’. https://doi.org/10.17605/OSF.IO/2TQNK ^44^

Data are available under the terms of the Creative Commons Attribution 4.0 International license (CC-BY 4.0).
