# Supplementary material for: An evaluation of the benefits and harms of antenatal corticosteroid treatment for women at risk of imminent preterm birth or prior to elective Caesarean-section: Study protocol for an individual participant data meta-analysis
Source: Wellcome Open Res. 2020 Feb 25;5:38. [Version 1] doi: 10.12688/wellcomeopenres.15661.1 (PMC7268149; doi:10.12688/wellcomeopenres.15661.1)
Supplement: Peer review history

# Peer review: Reviewer response for version 1

**DOI:** 10.21956/wellcomeopenres.17160.r38530

**Published:** 2020-06-02

**Authors:** Waldemar A. Carlo

**Affiliations:** 1 Department of Pediatrics, The University of Alabama at Birmingham, Birmingham, AL, USA

**Summary** 

 This manuscript is the study protocol for a planned individual participant meta-analysis (IPD-MA) of antenatal corticosteroid treatment for women at risk of imminent preterm birth or prior to elective Caesarean section (CS). Antenatal corticosteroid therapy (ACT) is one of the most beneficial therapies for threatened preterm labor and possibly other perinatal causes but many infants who may not benefit much are exposed and may have side effects. There are abundant data from RCTs. An IPD-MA is an ideal way to sort out the subgroups of mothers whose infants may benefit or not from exposure to ACT.

 The protocol is well designed and addresses several controversies in ACT including ACT given at >34 weeks gestation, ACT given prior to planned Caesarean-section, ACT given when the time from the previous ACT has been >7 days, and ACT given to those who deliver at term 

**Plain language summary**

 It is stated: “Antenatal corticosteroid treatment (ACT) has long been used to help reduce respiratory problems and neonatal death and is a cornerstone of treatment when preterm birth (defined as birth at <37 weeks gestation) is planned or expected, or when Caesarean-section at any gestation, is planned.” I would not consider that ACT “has long been used” or is “a cornerstone of treatment” for preterm labor at 34 to 36 weeks of for CS. These indications are more recent. The benefits of ACT in these infants are generally less consequential (no reduction in perinatal or neonatal deaths) and more marginal for RDS. Furthermore, harms have been reported, and there is more controversy on the use of ACT in these populations.

**Introduction** 

 This is an important IPD-MA as subgroups analyses are needed to be able to ascertain better the benefits and harms of ACT for specific patient subgroups and to better individualize patient care.

 Several subgroup analyses are planned. The four subgroups of studies when ACT is given >34 weeks gestation, when ACT is given prior to planned Caesarean-section, when ACT is given when the time from a previous ACT is >7 days, and when ACT given to those who deliver at term are important and controversial indications that an IPD-MA can address well.

 A subgroup IPD-MA that would be so important but is not considered would be by gestational age between 26 and 34 weeks of birth. It would be ideal to use an IPD-MA to determine the association of ACT and serious outcomes such as death at each week of gestation. The observational study by Travers *et al. *suggest that there is more than a 10-fold differences between in neonatal mortality in this wide range of gestational ages. Important benefits may not occur at gestational ages close to 34 weeks. Furthermore, these data are not by gestational age of treatment and are prone to bias as they are from an observational study. The suggestion is that this subgroup be added to the IPD-MA planned or a subsequent IPD-MA is performed on the subject. This is something I suspect the Co-opt working group has considered.

**Methods and Design**

 The study design is appropriate for the research questions. The authors use the PRISMA reporting guidelines to assure that the study will meet these specific guidelines.

 It is not clear if the subgroup analyses of the studies of ACT at >34 weeks will include an analysis at each week of gestation. Outcomes differ markedly at 34, 35, and 36 weeks, and this will be better addressed in an IPD-MA than in the individual RCTs.

 For the planned analyses, GA when first course of ACT given (<23 weeks, 23–28 weeks, 28–32 weeks, 32–34 weeks, 34–36 weeks, >36 weeks), it would be preferable to analyze the data by each week of gestation. If this is not possible, smoothing could be used by using three GAs at a time and moving over a week at a time.

 There is evidence that benefits may occur at much less than 48 hours post ACT. The planned subgroup analyses are for: length of time from administration of first dose of ACT until birth (<48 hours, 48 hours-7 days, 7–14 days, >14 days). It would be ideal to also use <24 hours as some emerging data indicate that most of the benefits can occur when birth is less than 24 hours after ACT.

Is the study design appropriate for the research question?

Yes

Is the rationale for, and objectives of, the study clearly described?

Yes

Are sufficient details of the methods provided to allow replication by others?

Yes

Are the datasets clearly presented in a useable and accessible format?

Yes

Reviewer Expertise:

I was the PI of the main trial and of the grant that funded the NEOPROM IPD-MA. Established current guidelines, initially published in 1995, recommend antenatal corticosteroids for mothers with preterm labor from 24 to 34 weeks' gestational age, but not before 24 weeks due to lack of data. However, many infants born before 24 weeks' gestation are provided intensive care.

I confirm that I have read this submission and believe that I have an appropriate level of expertise to confirm that it is of an acceptable scientific standard.

# Peer review: Reviewer response for version 1

**DOI:** 10.21956/wellcomeopenres.17160.r38016

**Published:** 2020-04-17

**Authors:** Martijn A. Oudijk

**Affiliations:** 1 Department of Obstetrics and Gynaecology, Amsterdam UMC, University of Amsterdam, Amsterdam, The Netherlands

This protocol for an IPD meta-analysis on the use of antenatal corticosteroids for threatened preterm birth of women planned for C section is set up well, and expected to provide the obstetric community with important answers regarding the effects of ACT. Especially the long term FU section is of the utmost importance.

 The group is expected to deliver this IPDMA, as they are all experts within this field, with excellent track records.

Is the study design appropriate for the research question?

Yes

Is the rationale for, and objectives of, the study clearly described?

Yes

Are sufficient details of the methods provided to allow replication by others?

Yes

Are the datasets clearly presented in a useable and accessible format?

Yes

Reviewer Expertise:

Preterm birth.

I confirm that I have read this submission and believe that I have an appropriate level of expertise to confirm that it is of an acceptable scientific standard.
